# Exploring the Photochemistry of an Ethyl Sinapate Dimer: An Attempt Toward a Better Ultraviolet Filter

**DOI:** 10.3389/fchem.2020.00633

**Published:** 2020-07-28

**Authors:** Michael D. Horbury, Matthew A. P. Turner, Jack S. Peters, Matthieu Mention, Amandine L. Flourat, Nicholas D. M. Hine, Florent Allais, Vasilios G. Stavros

**Affiliations:** ^1^School of Electronic and Electrical Engineering, University of Leeds, Leeds, United Kingdom; ^2^Department of Chemistry, University of Warwick, Coventry, United Kingdom; ^3^Physical and Theoretical Chemistry Laboratory, Department of Chemistry, University of Oxford, Oxford, United Kingdom; ^4^URD ABI, CEBB, AgroParisTech, Pomacle, France

**Keywords:** photoprotection, femtosecond, sinapates, photochemistry, spectroscopy

## Abstract

The photochemistry and photostability of a potential ultraviolet (UV) radiation filter, dehydrodiethylsinapate, with a broad absorption in the UVA region, is explored utilizing a combination of femtosecond time-resolved spectroscopy and steady-state irradiation studies. The time-resolved measurements show that this UV filter candidate undergoes excited state relaxation after UV absorption on a timescale of ~10 picoseconds, suggesting efficient relaxation. However, steady-state irradiation measurements show degradation under prolonged UV exposure. From a photochemical standpoint, this highlights the importance of considering both the ultrafast and “ultraslow” timescales when designing new potential UV filters.

## Introduction

In recent years, several artificial ultraviolet (UV) filters, serving the purpose of providing a front-line defense to UV radiation exposure, used in commercial sunscreen formulations, have come under scrutiny due to concerns about their safety (Saija et al., 2000; Matsui et al., 2009; Burnett and Wang, 2011; Loden et al., 2011; Afonso et al., 2014; Skotarczak et al., 2015; Sharma et al., 2017). Alongside this “sunscreen controversy,” incidences of skin cancer are on the rise (Stavros, 2014), even with increasing use of sunscreen formulations. This highlights the need for, not only improved education on general sun exposure and how to apply sunscreen formulas, but also a requisite for improved formulations containing safer UV filters. These new UV filters need to provide enhanced photoprotection along with being non-toxic, particularly when exposed to UV radiation.

One of the approaches to tackling these issues has been to study the intrinsic properties of photoprotective molecules found throughout nature (Saewan and Jimtaisong, 2015), which has had a few billion years head start in UV photoprotection. To this end, nature-based UV filters found in plants have garnered considerable interest as a starting point. One such molecule is the sinapate ester, sinapoyl malate. Indeed, a structurally related cinnamate, ethylhexyl methoxycinnamate, has already been employed as an artificial UV filter. However this has been recently shown to be genotoxic (Sharma et al., 2017), highlighting the urgent need for new non-toxic UV filters. The photochemistry responsible for sinapoyl malate's photoprotective capabilities has been proposed, along with that for related sinapate esters (Dean et al., 2014; Baker et al., 2016; Horbury et al., 2017a, 2018; Luo et al., 2017; Zhao et al., 2020). These studies have shown that, upon absorption of UV, these sinapate esters undergo an effective and ultrafast (femto- to picosecond, 10^−15^ and 10^−12^ s, respectively) *trans*-to-*cis* and *cis*-to-*trans* photoisomerization, which is responsible for their apparent long-term photostability and photoprotective nature. Therefore, gaining an understanding of the initial light/matter interaction which drives the overall photochemistry of the molecule can provide powerful insight in the development of new UV filters and there long-term photostability. Additionally, these sinapate esters possess strong absorptions in the UVA (400–315 nm) and in the UVB region (315–280 nm) of the solar spectrum.

**Figure 1:**
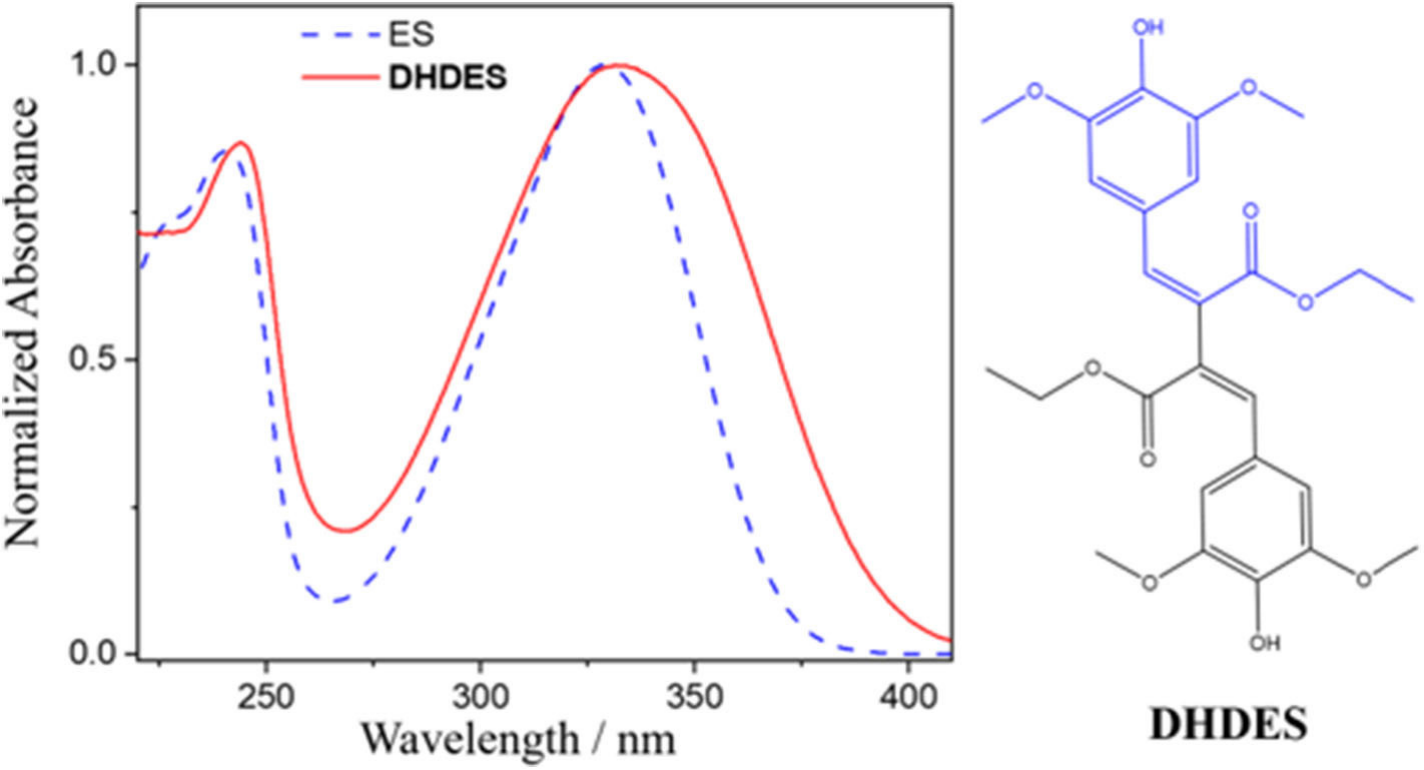
Normalized UV/Vis spectrum of **DHDES** (red) in ethanol. Additionally, the UV/Vis spectrum of ethyl-sinapate (ES) is overlaid (blue dashed line). The chemical structures of **DHDES** is shown right; chemical structure of ES is shown as blue in the structure of **DHDES**.

While these sinapate esters strongly absorb in the UVA, they do not cover the entirety of the UVA spectrum, i.e., sinapoyl malate's UVA absorption cuts off around 360 nm (Baker et al., 2016), which is particularly pertinent as this spectral region is linked to premature skin aging (Berneburg et al., 2000). Therefore, and ideally, if their absorption can be broadened across the entire UVA spectrum, it provides the means to a superior UV filter, spanning both the UVB and UVA. However, it is worth noting that increasing the UVA absorption does not imply a better UV filter; the molecule still needs to display a high level of photostability. One simple method of broadening, as well as spectrally red-shifting the absorption of these sinapate esters, is to increase the extent of conjugation in the π-system of the chromophore unit. An intuitive starting point is to add functionalization to the acrylic double bond. This was recently shown to be a promising approach by the addition of an identical ester group being added to the double bond of ethyl sinapate (shown in blue in Figure 1), which resulted in a redshift of the ground state absorption spectrum along with an increase in photostability (Horbury et al., 2019). We therefore utilize this approach, again, this time by dimerizing ethyl sinapate (Neudörffer et al., 2004). Ethyl sinapate has been shown to display very similar intrinsic properties to the plant UV filter, sinapoyl malate (Horbury et al., 2018). The resultant dimer is dehydrodiethylsinapate (**DHDES**, see Figure 1). As anticipated, **DHDES** possess a strong absorption across the (almost) entirety of the UVA region (Figure 1).

Whether the *structural* variant of sinapoyl malate, **DHDES**, with its enhanced UVA absorption, possesses the photostability (*dynamics*) displayed by sinapoyl malate and ethyl sinapate is the central question we seek to address in this work. Allied to this is the emerging trends that we may obtain through this work. Therefore, we investigate the photochemistry (*dynamics*) of **DHDES**, as a potential starting point for future nature-inspired UV filters with enhanced *function*; broader UVA coverage and efficient excitation-recovery cycles. To implement our *structure*-*dynamics*-*function* approach, we utilize femtosecond (fs) transient electronic (UV/visible, abbreviated to UV/Vis henceforth) absorption spectroscopy (TEAS) which has already provided valuable insight into the photochemistry of sinapate esters and related cinnamates (Vengris et al., 2005; Baker et al., 2016; Horbury et al., 2016, 2017a,b, 2018; Zhao et al., 2020). Additionally, the long-term photostability of this molecule was assessed *via* steady-state UV irradiation, monitored by UV/Vis absorption and ^1^H NMR spectroscopy. The experiments are complemented by computational results based on (time-dependant) density functional theory.

## Materials and Methods

### Transient Electronic Absorption Spectroscopy

The fs TEAS setup used to explore the photochemistry and photophysics of **DHDES** has been described in detail previously (Greenough et al., 2014a,b), however, information specific to the present experiments is provided herein. Samples of **DHDES** were made to a concentration of 1 mM in ethanol (absolute, VWR), ethylene glycol (technical, Arcos Organics), and glycerol (99.93%, Fisher. The fs pump pulses were generated by an optical parametric amplifier (TOPAS-C, Spectra-Physics) with a fluence of 200–800 μJ·cm^−2^. The pump excitation wavelength used was 332 nm for **DHDES** in ethanol, ethylene glycol, and glycerol; this wavelength correspond to the associated UV absorption maxima. The probe pulse was a broadband white light supercontinuum generated in a CaF_2_ window with a thickness of 2 mm, providing a probe spectral window of 330–675 nm. The pump-probe time delay (Δ*t*) was varied by adjusting the optical delay of the probe pulse, the maximum obtainable Δ*t* was 2 nanoseconds (ns). Changes in the optical density (ΔOD) of the samples were calculated from transmitted probe intensities, collected using a spectrometer (Avantes, AvaSpec-ULS1650F). The sample delivery system was a flow-through cell (Demountable Liquid Cell by Harrick Scientific Products Inc.) consisting of two CaF_2_ windows with a thickness of 1 mm for the front window and 2 mm for the back window, except in the case of **DHDES** in glycerol, where both windows were 2 mm thick due to the build-up pressure in the flow system (owing to glycerol's viscosity). The windows were spaced 100 μm apart to limit temporal dispersion of the pump and probe pulses. The sample was circulated using a diaphragm pump (SIMDOS, KNF) recirculating sample from a 25 mL reservoir to provide each pulse-pair with fresh sample.

### Steady-State Difference Spectra

Steady-state UV/Vis absorption spectra of **DHDES** in ethanol, were collected to determine long-term photostability. The sample was irradiated with an arc lamp (Fluorolog 3, Horiba) for up to 2 h, with the UV/Vis spectra taken at various time points, at the corresponding TEAS excitation wavelength, using an 8 nm bandwidth of the irradiation source. The fluence used during irradiation of **DHDES** was set to 100–200 μJ·cm^−2^ to mimic solar incidence conditions. The UV/Vis spectra were measured using a UV/Vis spectrometer (Cary 60, Agilent Technologies).

### Computational Calculations

The structure of **DHDES** was generated in its *cis*,*cis*-isomer and *trans*,*trans*-isomer (see Figures 1, **4**), as well as a contorted *cis*,*trans*-isomer, using VMD (Humphrey et al., 1996) with the molefacture plugin. Each of these structures underwent a density functional theory (DFT) geometry optimization with a cc-pVTZ basis set (Dunning, 1989) and the PBE0 functional (Adamo and Barone, 1999), using the NWChem software (Valiev et al., 2010). The *cis*,*trans*-isomer of **DHDES** was significantly energetically disfavored with respect to the other two isomers, and the geometry optimization did not converge with a reasonable level of convergence criteria; therefore this isomer was discounted from further study. Following relaxation, linear-response time-dependant DFT calculations were conducted at the optimized ground state geometries of the *cis*,*cis*-isomer and *trans,trans*-isomer to obtain their optical absorption spectra. Again, the level of theory was cc-pVTZ/PBE0 and the NWChem software was employed. In all calculations, the conductor-like screening model (COSMO) was used to approximate the effect of the solvent (Klamt and Schüürmann, 1993; York and Karplus, 1999). The default COSMO solvent model for ethanol within NWChem was used, the descriptors of which are based on the Minnesota Solvent Descriptor Database (Winget et al., 1999).

### Synthetic Procedures and ^1^H NMR

**DHDES** and **Me-DHDES** were synthesized using the procedure published by Mention et al. (2020) ^1^H NMR spectra of **DHDES** and **Me-DHDES** (we discuss the reason for studying this system below) in CDCl_3_ were recorded at 300 MHz on a Bruker Fourier 300, pre- and post-irradiation using a Rayonet RPR-200 after irradiation of 60 min at 302 nm; this wavelength was used due to the limited spectral choices of the Rayonet RPR-200.

### Fitting

To retrieve the dynamical information contained within the transient absorption spectra, a sequential $\left(A\overset{\tau_{1}}{\to }B\overset{\tau_{2}}{\to }C\overset{\tau_{3}}{\to }D\right)$ global (across all wavelengths 330–675 nm) fitting technique was performed, using the software package Glotaran (Mullen and Van Stokkum, 2007; Snellenburg et al., 2012). The transient absorption spectra of **DHDES** were fit using four time-constants (τ_*n*_, where *n* = 1–4). Each time-constant is linked to an evolution associated difference spectrum (EADS) that represents the evolving spectral features related to that time-constant. All fits were convoluted with a Gaussian function to model our instrument response function (~80 fs, for glycerol ~100 fs). The final time-constant used in our model (τ_4_ for **DHDES**) accounts for the long-lived photoproduct; this time-constant is reported to be >> 2 ns.

## Results

### Transient Electronic Absorption Spectroscopy

**Figure 2:**
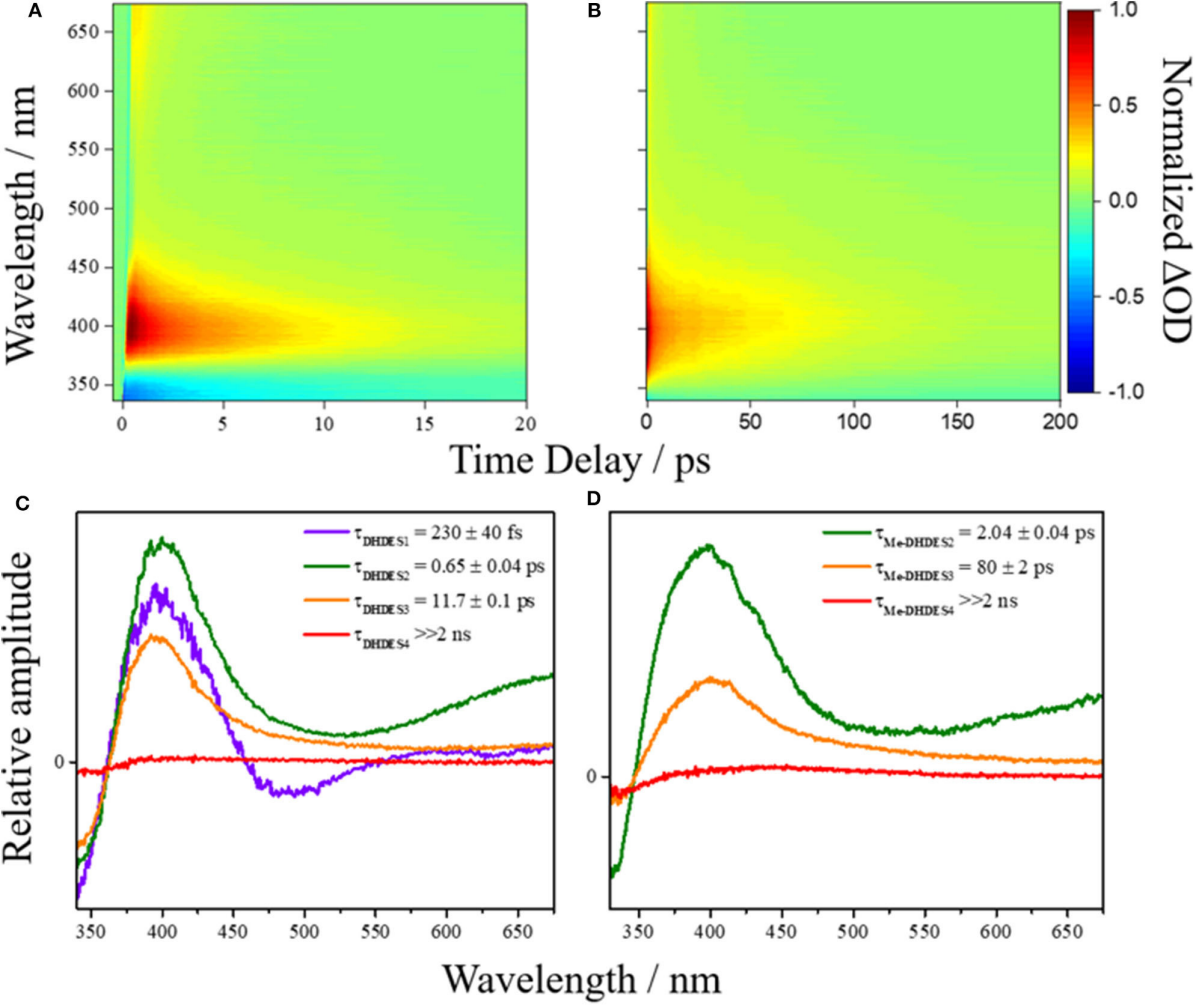
False colormap of the TAS of **(A) DHDES** and **(B) Me-DHDES** in ethanol, with intensity representing normalized change in optical density (OD). Additionally, the EADS produced by the sequential global fitting of the TAS are also displayed for **(C) DHDES** and **(D)** Me-DHDES.

First, we consider the resulting transient absorption spectra of **DHDES** in ethanol shown in Figure 2A (see ESI Figure S1 for transient absorption spectra of **DHDES** in ethylene glycol and glycerol). Note that the related **Me-DHDES**, a methylated version of **DHDES**, is also shown in Figure 2B, but will be discussed later. After initial photoexcitation at 332 nm, the transient absorption spectrum consist of three distinct spectral features, the first being a ground state bleach centered at ~340 nm, the second is a large excited state absorption at ~400 nm and the third is a smaller excited state absorption at ~675 nm. As Δ*t* increases, the excited state absorption at 675 nm begins to decay, leaving a broad flat absorption alongside the large excited state absorption feature at 400 nm. At longer Δ*t*, the ground state bleach and both excited state absorption features tend toward the baseline. Once the excited state absorption features have completely decayed away, a new absorption feature (not immediately evident) spanning 390–600 nm is revealed. Some residual ground state bleach at 340 nm remains. Both features persist beyond the maximum Δ*t* (see ESI Figure S2 for transient absorption spectrum at Δ*t* = 2 ns).

The time-constants produced from fitting the transient absorption spectra are provided in Table 1, and the corresponding EADS are shown in Figures 2C,D (see ESI Figure S3 for additional EADS for **DHDES** in ethylene glycol and glycerol, along with residuals Figures S4–S8 for **DHDES** in ethanol, ethylene glycol, glycerol, and **Me-DHDES** in ethanol, respectively).

**Table 1:** The values of the time-constants (τ_xN_, where x represents either **DHDES** or **Me-DHDES**) provided from globally fitting the transient absorption spectra of **DHDES** in ethanol, ethylene glycol, and glycerol, **Me-DHDES** in ethanol.

	**τ_**x1**_**	**τ_**x2**_**	**τ_**x3**_**
**DHDES**			
Ethanol	230 ± 40 fs	0.65 ± 0.04 ps	11.7 ± 0.1 ps
Ethylene Glycol	270 ± 40 fs	1.3 ± 0.1 ps	12.5 ± 0.2 ps
Glycerol	100 ± 50 fs	1.36 ± 0.05 ps	39.3 ± 0.4 ps
**Me-DHDES**	n/a	2.04 ± 0.04 ps	80 ± 2 ps

*Errors are quoted to 2σ except when an error returned was less than half the instrument response. In such cases, the error is reported as half the instrument response. For fit residuals, see ESI Figures S4–S7*.

### Steady-State Irradiation

**Figure 3:**
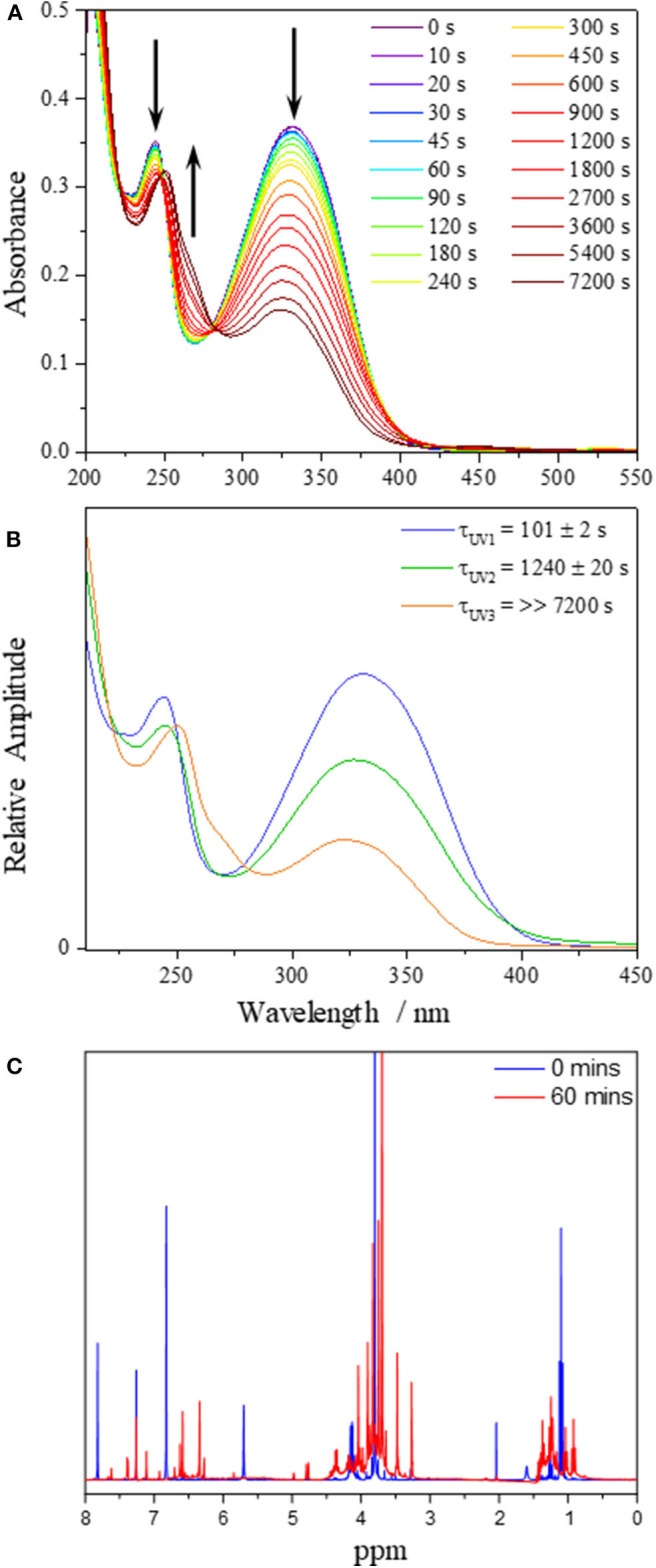
**(A)** UV/Vis spectra of **DHDES** in ethanol taken at various time points during irradiation at 332 nm over 7,200 s. **(B)** EAS from the sequential global fit of the data displayed in **(A)**. **(C)** ^1^H NMR spectra of **DHDES** pre (blue) and post (red) irradiation in ethanol.

Steady-state irradiation of **DHDES** in ethanol was carried out to determine its long-term photostability under constant UV exposure. To monitor any changes to the sample, both UV/Vis (Figure 3A as well as extracted evolution associated spectra, EAS, Figure 3B) and ^1^H NMR (Figure 3C and Figure S9) spectra were recorded at various time points of irradiation. Similar experiments were carried out for **Me-DHDES** (see ESI Figures S10, S11 for further details).

The evolution of the UV/Vis spectra during irradiation for **DHDES** in ethanol, is shown in Figure 3A and clearly indicates that the main absorption feature spanning the UVA and UVB regions of the absorption spectrum undergoes a significant decay (~60% reduction in 2 h). However, this decay appears to consist of two distinct spectral evolutions, as represented by the arrows in Figure 3A; a decay to yield (existing) features at ~330 and ~240 nm, and a growth of new absorption features at ~250 and ~270 nm.

To better distinguish the processes that are occurring, we performed a sequential global (210–450 nm) fit of the UV/Vis spectra. The resulting EAS and time-constants are shown in Figure 3B. Cursory comparison of EAS(τ_UV1_) (101 ± 2 s) with EAS(τ_UV2_) (1240 ± 20 s) suggest that upon irradiation of **DHDES**, the absorption intensity drops. Under prolonged irradiation the spectra continue to show a decrease in absorption; however, additional spectral features begin to grow in. These new absorption features consist of a peak at ~270 nm and a marked red-shift of the peak at ~240 nm to ~250 nm as seen in EAS(τ_UV3_) (>>7,200 s). This is very likely indicative of a new species being generated.

To identify any newly generated species after UV radiation exposure, a ^1^H NMR spectrum of **DHDES** was taken before and after 60 min irradiation (see Figure 3C). One can observe that the peaks corresponding to the benzene ring protons of **DHDES** (6.78 and 7.83 ppm) have disappeared and peaks appear at ~6.4 and ~6.6 ppm, along with many new peaks in the 3.5–5.0 ppm as well as the 0.7–1.5 ppm regions, demonstrating the transformation of the latter into multiple new compounds under radiation (Figure 3C). The appearance of broad peaks in the ^1^H NMR spectrum after irradiation hints at the formation of oligomers. Additionally it is likely that **DHDES** is undergoing an esterification with ethanol when exposed to UV; this has previously been seen with 1,4-diphenyl-1,3-dibutene (Saltiel and Redwood, 2016). We will return to discuss another potential product, a tricyclic compound, in the discussion (*vide infra*).

### Computational Results

**Figure 4:**
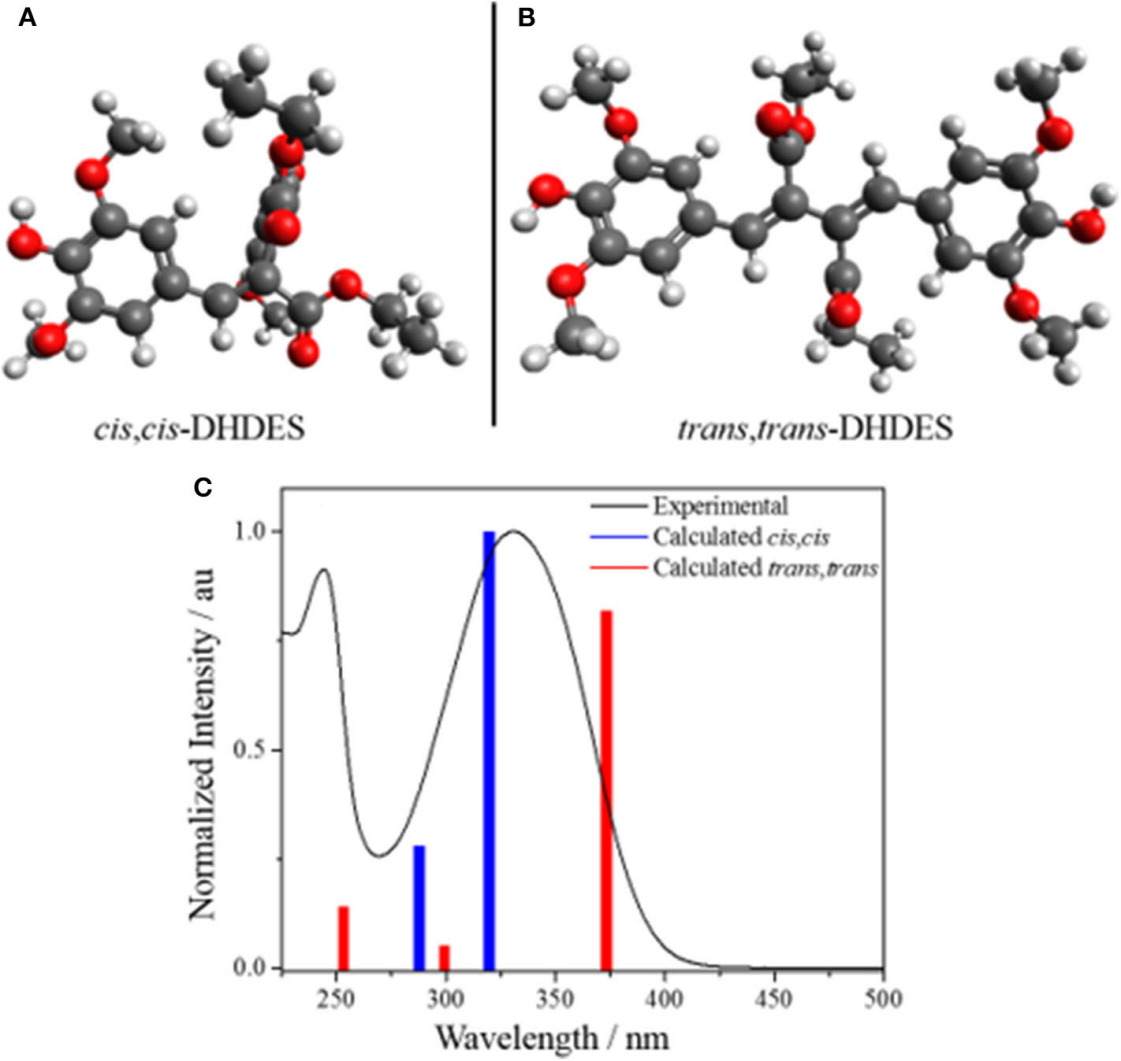
Geometry calculations of the stable ground state isomers **(A)** *cis,cis*-**DHDES** and **(B)** *trans,trans*-**DHDES. (C)** Predicted UV/Vis stick spectra (*cis,cis*-**DHDES** blue bars, *trans,trans*-**DHDES** red bars) overlaid with experimental UV/Vis spectrum **of DHDES** in ethanol (black line).

The calculated ground state isomers of two stable isomers of **DHDES** (*cis*,*cis*- and *trans*,*trans*-isomers), are shown in Figure 4 (*NB* the *trans,trans*-isomer of **DHDES** is also show in in Figure 1). The *cis*,*cis*-isomer has the lower energy of the two conformers with the *trans*,*trans*-isomer being ~0.3 eV higher in energy. A molecule of this size likely has a complicated ground-state energy surface with multiple local minima corresponding to different stable geometries. As such, it is challenging to determine that the structure chosen for both conformers is the global minimum energy structures and, indeed, it is likely multiple structures exist in solution. Owing to this, the predicted energies for these species, as well as predicted vertical excitation energies, are presented as an approximation of each form rather than as quantitative data. Furthermore, the effect of explicit solvent interactions are not captured by our technique, these likely also have an effect on the relative energies of the species (Zuehlsdorff et al., 2017; Turner et al., 2019). Additionally, the two isomers display several significant differences in geometry, beyond just isomerization around the double bonds. In the case of the *cis, cis*-isomer, the two ethyl sinapate subunits are at ~90° to each other, which hinders conjugation between the two ethyl sinapate subunits. It should be noted that a planar *cis*,*cis*-**DHDES** is sterically unfavorable, as several atoms would need to occupy the same space. However, the *trans*,*trans*-isomer is *almost* planar (~24° twist between the two benzene rings), but the two ethyl sinapate subunits are sufficiently twisted relative to one another to weaken the extended conjugation across both subunits. This lack of conjugation is further supported by the UV/Vis spectra of **DHDES** (Figure 1); if there was extended conjugation across the entire molecule, one would anticipate a starker red-shift in the absorption.

In addition to the calculated geometries, UV/Vis spectra for the *cis*,*cis*-**DHDES** and *trans*,*trans*-**DHDES** were calculated, and also shown in Figure 4. The spectra show the main absorption peak for *cis*,*cis*-**DHDES** is at 322 nm, while for *trans*,*trans*-**DHDES** it is at 371 nm; the *cis*,*cis*-isomer has a higher oscillator strength. Due to the calculated peaks for both isomers being within the experimental absorption peak, it possible that our sample of **DHDES** contains both *cis*,*cis*- and *trans*,*trans*-isomers, which in turn gives rise to the increased broadness in absorption compared to ethyl sinapate (see Figure 1). While a calculated energy difference of 0.3 eV would suggest that the *cis,cis*-isomer would be heavily favored, it is possible that the calculated energy difference would be reduced if an explicit solvent model was used. Furthermore, we believe that during the synthesis of **DHDES** both isomers are generated.

## Discussion

We now consider the ultrafast photochemistry and photophysics of **DHDES**. As discussed *supra*, due to the lack of conjugation, a consequence of the twisted geometries between the two ethyl sinapate substructures (see Figures 4A,B), one would anticipate that initial photoexcitation would yield a transient absorption spectrum analogous to the transient absorption spectrum of ethyl sinapate in ethanol. Whilst this is not immediately apparent in the measured transient absorption spectra of **DHDES**, the EADS(τ_DHDES1_) (230 ± 40 fs) in ethanol is mildly similar to the first EADS of ethyl sinapate in ethanol (see ESI Figures S12, S13 for more details). We therefore assign the first time-constant τ_DHDES1_, and its corresponding EADS(τ_DHDES1_), to the **DHDES** undergoing a molecular motion that allows for increased coupling between the two ethyl sinapate substructures, thus the decay of the ethyl sinapate-like features; this is substantiated by the significant change between EADS(τ_DHDES1_) and EADS(τ_DHDES2_) (0.65 ± 0.04 ps), see Figure 2C. With the decay of the ethyl sinapate-like features, the comparison of **DHDES** to ethyl sinapate now ends.

Next, we consider the time-constant τ_**DHDES**2_ and its corresponding EADS(τ_**DHDES**2_). The EADS(τ_DHDES2_) is dominated by two large excited state absorption features at ~400 and ~675 nm, which we attribute to a specific **DHDES** isomer (see Figure 4). We draw confidence here, by noting that the excited state absorption feature at ~675 nm is like the excited state absorption seen in TEAS recorded for 1,4-disyringol-1,3-butadiene (DSB, see ESI Figures S14, S15). DSB is the backbone structure of **DHDES**, which remains planar, unlike **DHDES**, due to the absence of the ester functionalities (see ESI Figure S16). Consequently, it is plausible that the absorption feature at 675 nm in **DHDES** is attributed to a planar form with increased conjugation, akin to DSB. Since *trans*,*trans*-**DHDES**, is near-planar in the ground state, this isomer serves as the leading candidate; the excess energy imparted by the absorption of a UV photon may allow *trans*,*trans*-**DHDES** to approach planarity, as excited state population samples the excited state potential energy landscape. While we cannot rule out that this is also happening in *cis*,*cis*-**DHDES**, one notes that this would require severe nuclear rearrangement, including *cis,cis*-to-*trans,trans* isomerization (*vide supra*), to even approach planarity. With this in mind, we suggest that the excited state absorption seen at 400 nm is likely attributed to the absorption from the *cis, cis*-**DHDES** electronic excited state. When comparing the EADS(τ_DHDES2_) and EADS(τ_DHDES3_) (11.7 ± 0.1 ps), the major difference is the absence of the excited state absorption at ~675 nm. Whether or not this is due to *trans*,*trans*-**DHDES**, relaxing back to the electronic ground-state on the timescale of τ_DHDES2_, is unknown. Unfortunately, due to the sample potentially consisting of both isomers of **DHDES**, the transient absorption spectra and EADS are heavily convoluted making it hard to distinguish which process is associated with which **DHDES** isomer.

Thirdly, the EADS(τ_DHDES3_) resembles the decay of the remaining excited state absorption at 400 nm. As this excited state absorption is attributed to the *cis*,*cis*-**DHDES** isomer (see above) it represents the decay of the electronically excited state of *cis*,*cis*-**DHDES**, but with the caveat that the *trans*,*trans*-**DHDES** could be present and also contributes to the EADS(τ_DHDES3_) and timescale. Finally, EADS(τ_DHDES4_) (>>2 ns), models the long-lived photoproduct (see ESI Figure S2 for transient absorption spectrum at Δ*t* = 2 ns). Whether this is due to a transient species such as a triplet state or a molecular photoproduct is unknown.

To help determine whether the electronic excited state relaxation, for either isomer, is mediated by a photoisomerization motion leading to a conical intersection between the electronic excited state and ground state, akin to ethyl sinapate (Horbury et al., 2018), additional TEAS measurements were performed in more viscous solvents: ethylene glycol (η = 21) (Tsierkezos and Molinou, 1998) and glycerol (η = 1,412) (Segur and Oberstar, 1951) (see ESI for transient absorption spectra, ethanol η = 1.19) (Khattab et al., 2012). From these measurements, we determined the viscosity dependence, α, of the time-constant τ_DHDES2_ and τ_DHDES3_, as these time-constant are linked to the decay of the electronic excited state, which may be mediated by photoisomerization (Espagne et al., 2006; Horbury et al., 2017a). The retrieved α value for τ_DHDES2_ was 0.09 ± 0.06 and for was τ_DHDES3_ 0.18 ± 0.07. The value returned for τ_DHDES2_ suggests that large amplitude nuclear motion linked to this time-constant is minor, meaning that either the *trans*,*trans*-**DHDES** undergoes very little (large amplitude) nuclear motion to relax or that this process involves a change in electronic states. While τ_DHDES3_ is a smaller value compared to related sinapate esters (~0.34) (Horbury et al., 2017a), it still suggests that the electronic excited state decay of **DHDES** involves some substantial nuclear motion, of which a photoisomerization could be a candidate.

**Scheme 1:**
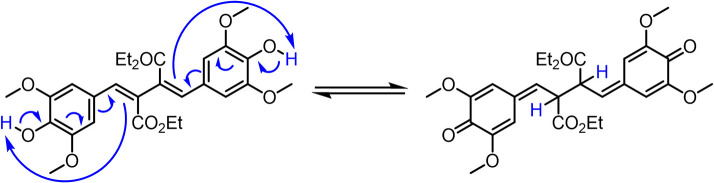
Generation of the quinone methide from **DHDES**.

The incomplete recovery of the ground state bleach suggests that (at least) a small fraction of **DHDES** is not returning to its original form. This incomplete recovery of the ground state bleach is not unexpected as steady-state irradiation has demonstrated **DHDES** degrades over persistent UV exposure. It has previously been reported that a quinone methide species can be formed upon electrochemically-induced oxidation and one could assume that a similar mechanism could also occur upon irradiation, this mechanism is shown in Scheme 1 (Neudörffer et al., 2006).

Another potential mechanism for the formation of the quinone methide is that it is initiated by O–H bond fission. While one would anticipate that this pathway would be blocked by the presence of an intramolecular hydrogen bond between the hydroxy and methoxy groups, previous studies on syringol, show it can undergo O–H bond fission. Owing to the non-planar geometry of syringol in the excited electronic state, this serves to weaken the intramolecular hydrogen bond sufficiently enough to allow for O–H bond fission to occur (Young et al., 2014). It is therefore not unreasonable to assume that the strained and twisted nature (see above) of **DHDES** induces a similar affect, by weakening the intramolecular hydrogen bonding, thus, opening up the O–H fission pathway along, say, a dissociative ^1^πσ^*^ state, in keeping with previous studies (Roberts and Stavros, 2014).

**Scheme 2:**
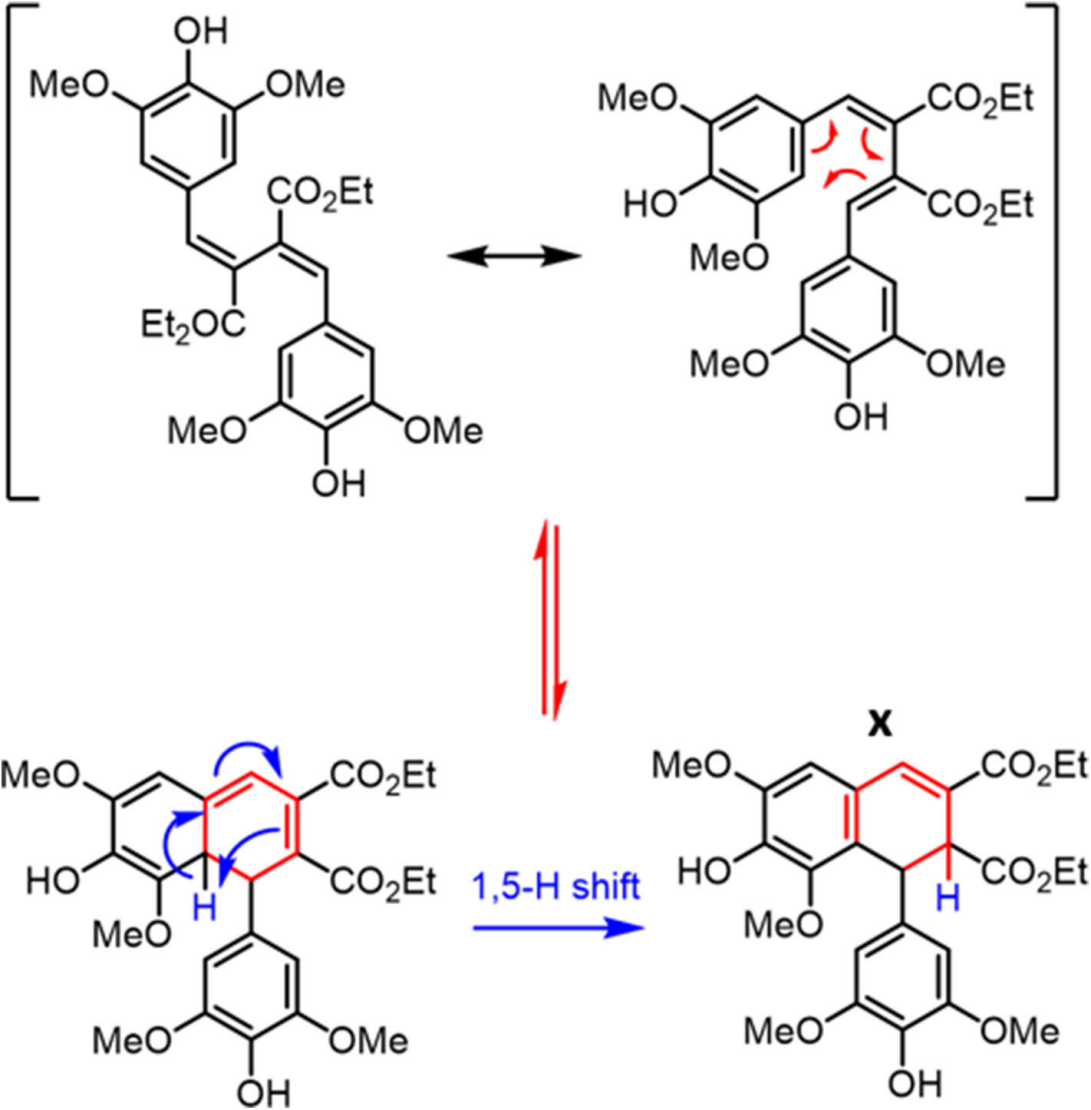
Generation of the tricyclic species **X** from **DHDES**.

To test such a hypothesis for O–H bond fission shown in Scheme 1, we have replaced O–H with O–CH_3_; previous studies in similar systems have shown that O–H fission is more facile over O–C fission. (Hadden et al., 2012) Therefore, methylation could lead to stabilization of **DHDES** under prolonged UV exposure. The methylated form of **DHDES**, termed **Me-DHDES**, in ethanol (1 mM), was then studied using TEAS (photoexcited at 310 nm) and steady-state absorption to see if the formation of the proposed quinonic methide species is prevented. The resulting transient absorption spectra and EADS are shown in Figures 2B,D. While the spectral features that are present in the transient absorption spectra match the ones seen in **DHDES**, the time-constants (see Table 1) clearly differ in two ways: (1) τ_DHDES1_ is absent; and (2) τ_Me−DHDES3_ is substantially longer than τ_DHDES3_ (80 ± 2 ps versus 12.5 ± 0.2 ps for **DHDES**). However, the EADS(τ_Me−DHDES2_) (2.04 ± 0.04 ps) and EADS(τ_Me−DHDES3_) are remarkably similar spectrally to those of **DHDES**. Therefore, we are confident that **Me-DHDES** undergoes the same relaxation mechanism as **DHDES**. Additionally, the steady-state UV/Vis spectra (see ESI Figure S10) taken during prolonged UV irradiation and the appearance of new proton peaks in the ^1^H NMR spectra taken pre- and post-irradiation (see ESI Figure S11), demonstrate **Me-DHDES** is also undergoing degradation. Since, the addition of the methoxy group is likely to supress bond-rearrangement proposed in Scheme 1, the revised degradation pathway is the formation of the tricyclic compound **X**, formed through a photo cyclo-addition followed by a re-aromatization via an 1,5-H shift (Scheme 2); this photoproduct would be expected to exhibit ^1^H NMR peaks at ~6.3 and ~6.6 ppm in Figure 3C. Consideration of the above and given the same proton peaks are present in the post-irradiation ^1^H-NMR spectra of **DHDES** and **Me-DHDES** (see ESI Figures S9, S11), the degradation pathway between the two is likely the same, this pathway is likely occurring alongside the photoinduced esterification with ethanol (*vide supra*).

## Conclusion

We have explored the photochemistry and photostability of a potential UV filter, dehydrodiethylsinapate (**DHDES**), which provides almost full absorption coverage of the UVA region of the electromagnetic spectrum. This was achieved using a combination of femtosecond transient electronic (UV/Vis) absorption spectroscopy (TEAS) and steady-state UV irradiation, monitored by both UV/Vis and ^1^H NMR spectroscopy.

The photodynamics observed in the TEAS measurements suggest **DHDES** undergoes an ultrafast electronic excited state relaxation, potentially mediated *via* a photoisomerization pathway. However, the steady-state irradiation studies demonstrate that **DHDES** is not photostable under prolonged UV exposure. We attempted to thwart degradation by protecting the O–H with O–Me without success. Through the result of this methylation in combination with ^1^H NMR, we have identified a potential cause of degradation, due to the formation of a tricyclic species. It is likely that the tricyclic species further degrades under UV exposure, leading to the formation of the myriad of unidentified molecular species seen in the post irradiation 1H NMR spectrum. Therefore, if one can by-pass the formation of this tricyclic species, this could expand the molecular diversity based around a core DHDES structure, potentially leading to promising next generation, broad-spectrum and nature-inspired, UV filter molecules.

Importantly, this work demonstrates that manipulating a molecules' structure, whilst trying to preserve the ultrafast dynamics, can have both positive and negative implications on its function. Indeed, we have shown that **DHDES** has enhanced UVA absorption, whilst still possessing a short-lived excited state. However, this is at the cost of long term photostability. Undeniably, what pervades the present study is that chemical intuition, increased conjugation and thus broader UVA absorption, does not necessarily result in a better (nature-inspired) UV filter. It is clear that in order to progress this structure-dynamics-function approach, we will need to seek increasing levels of guidance from theory, a current strategy underway in our laboratory, in our search for next generation, nature inspired UV filters which could be included in commercial sunscreen formulations.

## Data Availability Statement

The datasets presented in this study can be found in online repositories. The names of the repository/repositories and accession number(s) can be found below: https://zenodo.org/record/3741408, doi: 10.5281/zenodo.3741408.

## Author Contributions

MH acquired and analyzed the time-resolved and steady-state spectroscopic data and prepared the manuscript. JP provided assistant in the acquisition of the time-resolved and steady-state spectroscopic data. AF, MM, and FA conceived and performed the syntheses, as well as contributing to the preparation of the manuscript. MT and NH performed and analyzed the computational calculations. VS conceived the experiments and provided guidance in data analysis and interpretation and the writing of the manuscript. All authors contributed to the article and approved the submitted version.

## Conflict of Interest

The authors declare that the research was conducted in the absence of any commercial or financial relationships that could be construed as a potential conflict of interest.
