# Supplementary material for: Exploring the Photochemistry of an Ethyl Sinapate Dimer: An Attempt Toward a Better Ultraviolet Filter
Source: Front Chem. 2020 Jul 28;8:633. doi: 10.3389/fchem.2020.00633 (PMC7399488; doi:10.3389/fchem.2020.00633)
Supplement: Supplementary file 1 [file Data_Sheet_1.DOCX]

Exploring the photochemistry of an ethyl sinapate dimer: An attempt towards a better ultraviolet filter

**Michael D. Horbury,^*1^ Matthew A. P. Turner,^2^ Jack S. Peters,^3^ Matthieu Mention,^4^ Amandine L. Flourat,^4^ Nicholas D. M. Hine,^2^ Florent Allais,^4^ and Vasilios G. Stavros*^2^**

^1^School of Electronic and Electrical Engineering, University of Leeds, Leeds, LS2 9JT ^2^Department of Chemistry, University of Warwick, Gibbet Hill, Coventry, CV4 7AL
^3^Chemistry Research Laboratory, 12 Mansfield Road, Oxford, OX1 3TA ^4^Chaire ABI–AgroParisTech, CEBB, 3 rue des Rouges Terres, 51110 Pomacle, France

# Additional Transient Electronic Absorption Spectroscopy Measurements in Ethylene Glycol and Glycerol


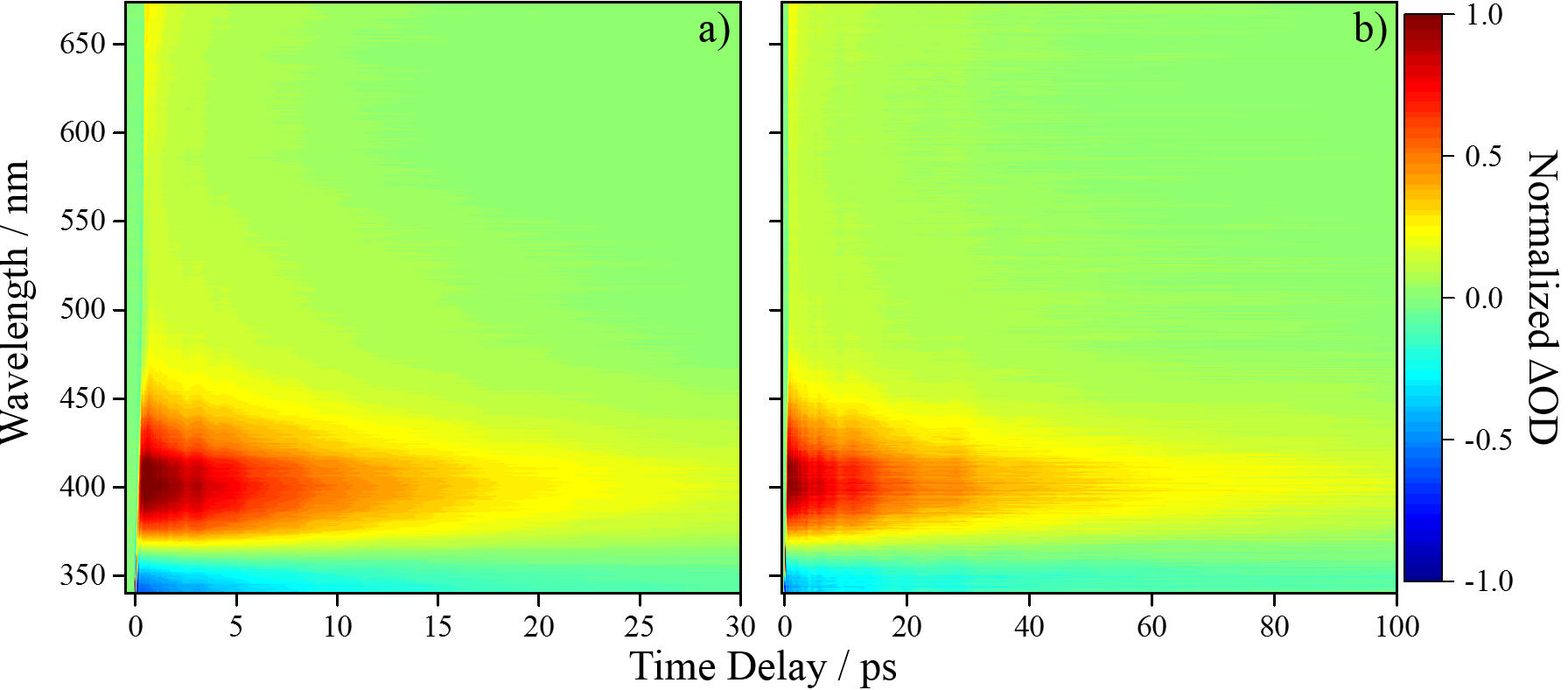


**Figure S1**: False colourmap of the transient absorption spectra of **DHDES** in a) ethylene glycol and b) glycerol, with intensity representing normalized change in optical density (ΔOD).

**Figure S2**: transient absorption spectra taken at a pump-probe time delay of 2 ns for **DHDES** in a) ethanol, b) ethylene glycol and c) glycerol.


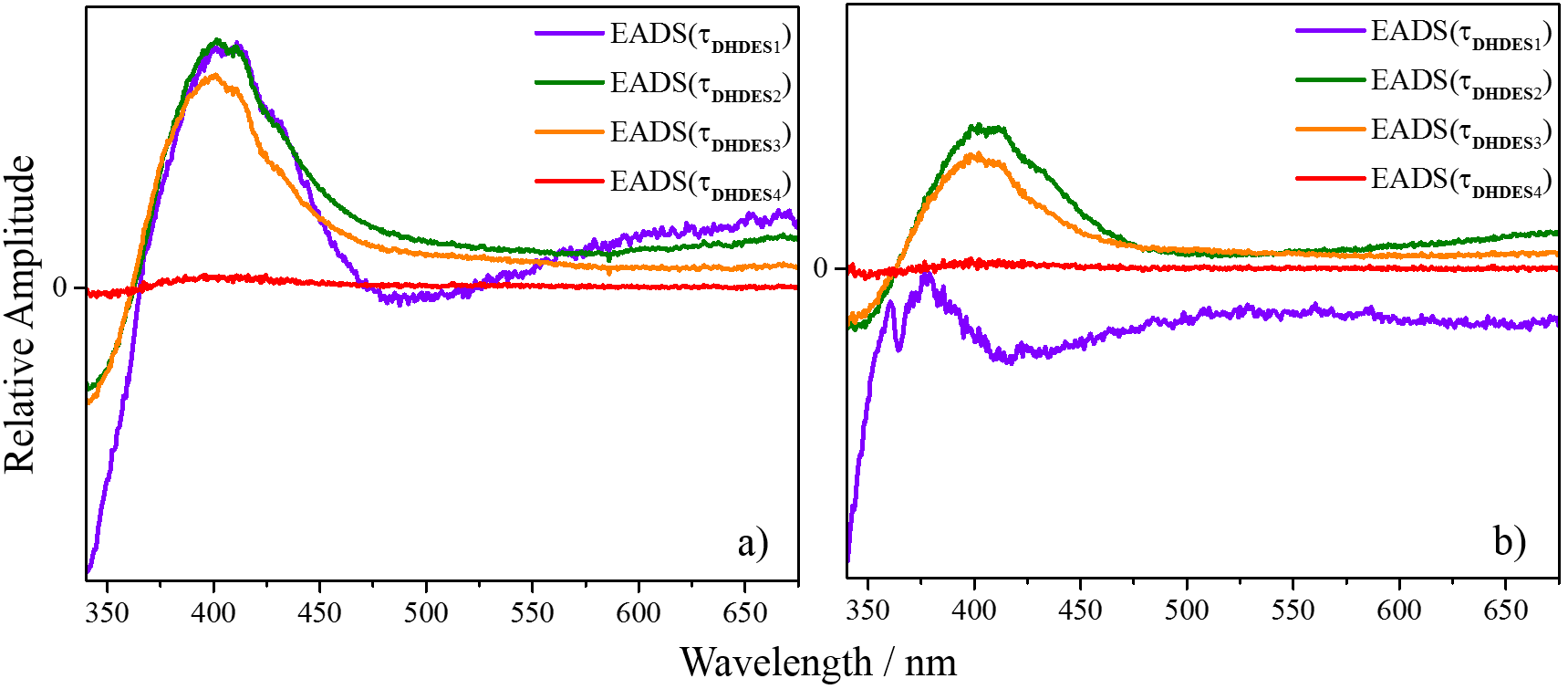


**Figure S3**: The EADS produced by the sequential global fitting of the transient absorption spectra for **DHDES** in a) ethylene glycol and b) glycerol. We note that EADS(τ**_DHDES_**_1_) for glycerol has a negative amplitude at all wavelengths, this is likely due to the corresponding lifetime been close to the instrument response of the system, leading to the EADS(τ**_DHDES_**_1_) being a convolution of the time-zero artifacts and the signal from **DHDES**.

# Fit Residuals


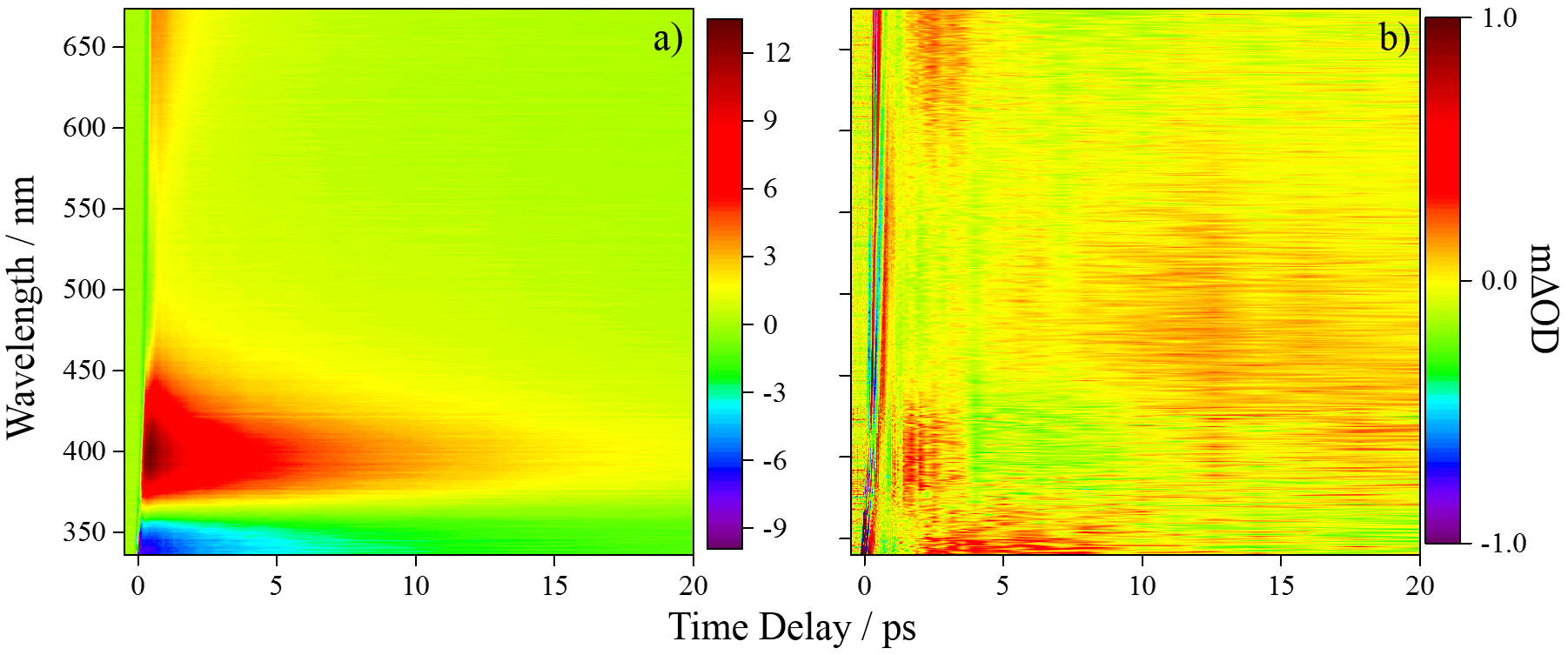


**Figure S4**: a) False colour map of non-normalized transient absorption spectra of **DHDES** in ethanol and b) the corresponding fit residual.


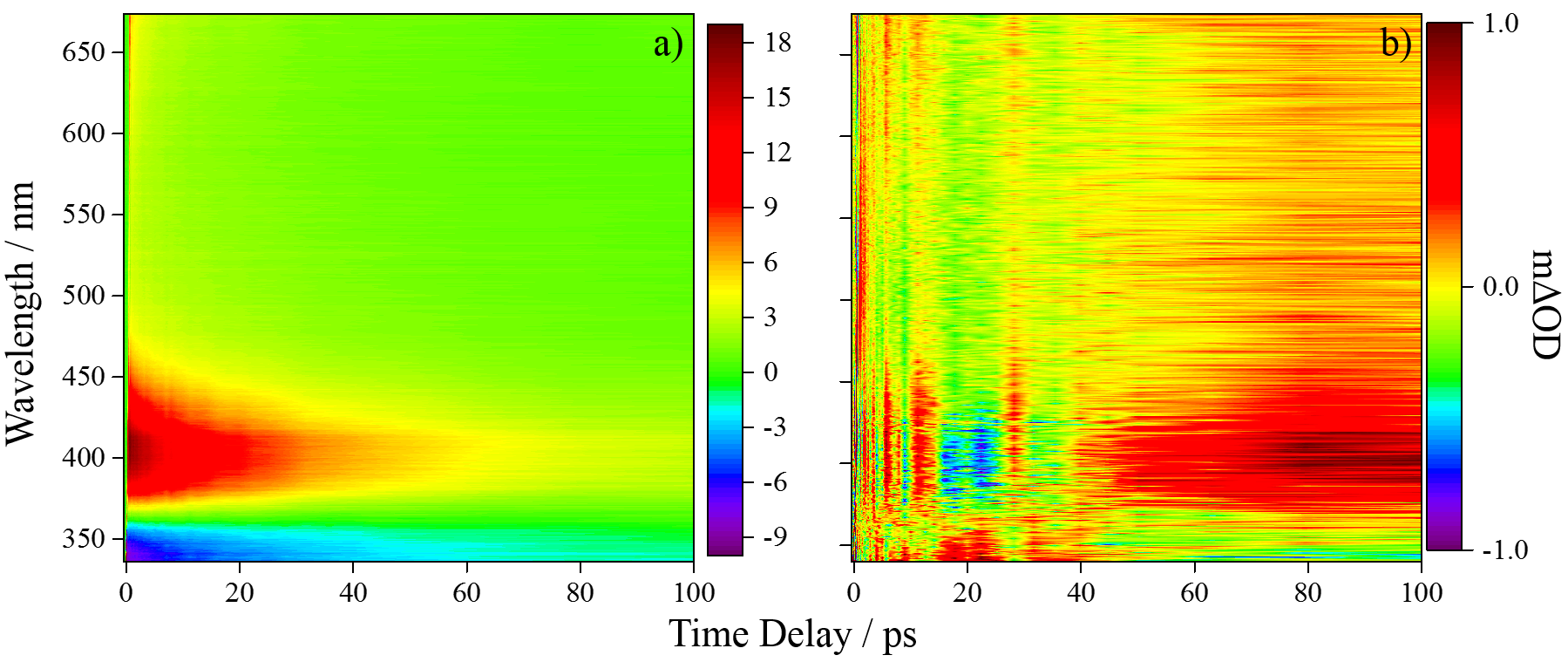


**Figure S6:** a) False colour map of non-normalized transient absorption spectra of **DHDES** in glycerol and b) the corresponding fit residual.


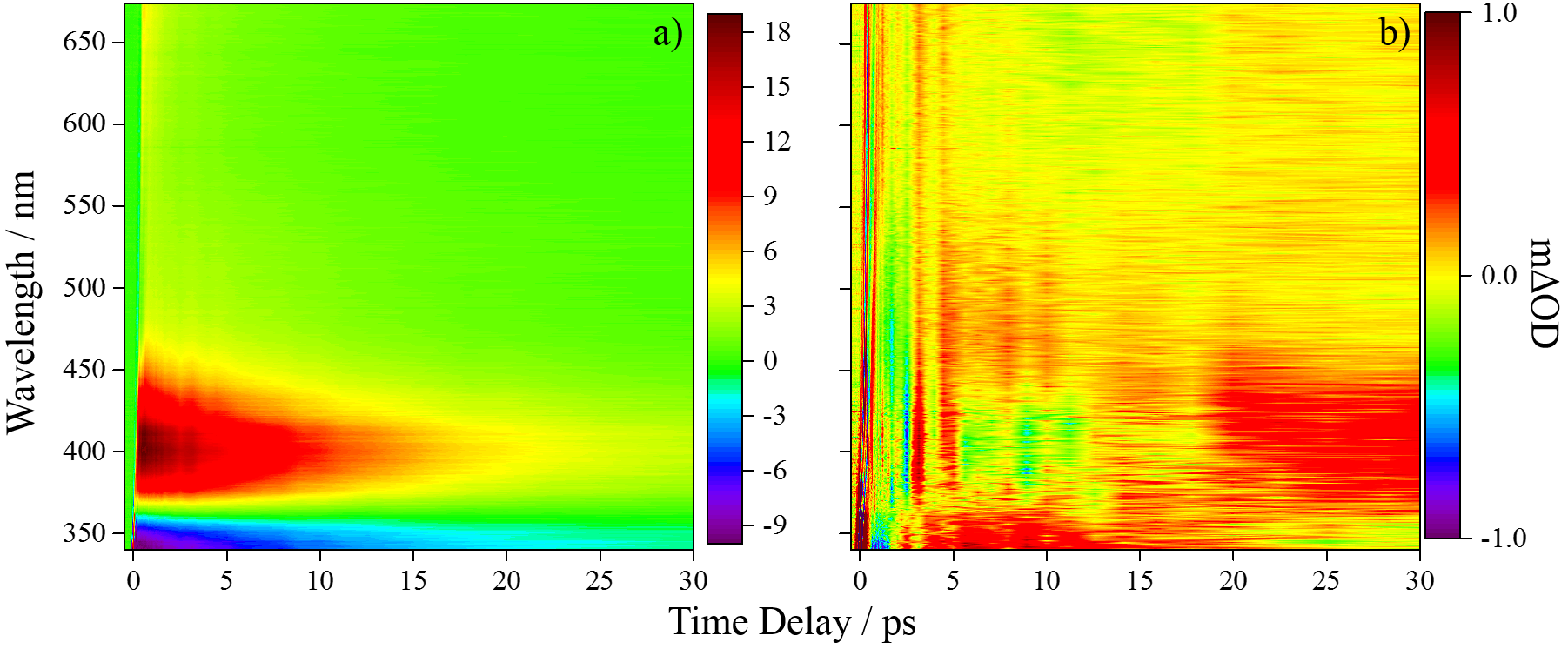


**Figure S5:** a) False colour map of non-normalized transient absorption spectra of **DHDES** in ethylene glycol and b) the corresponding fit residual.

**Figure S8**: Transient absorption spectra of **DHDES** in a) ethanol, b) ethylene glycol and c) glycerol and **Me-DHDES** in d) ethanol.


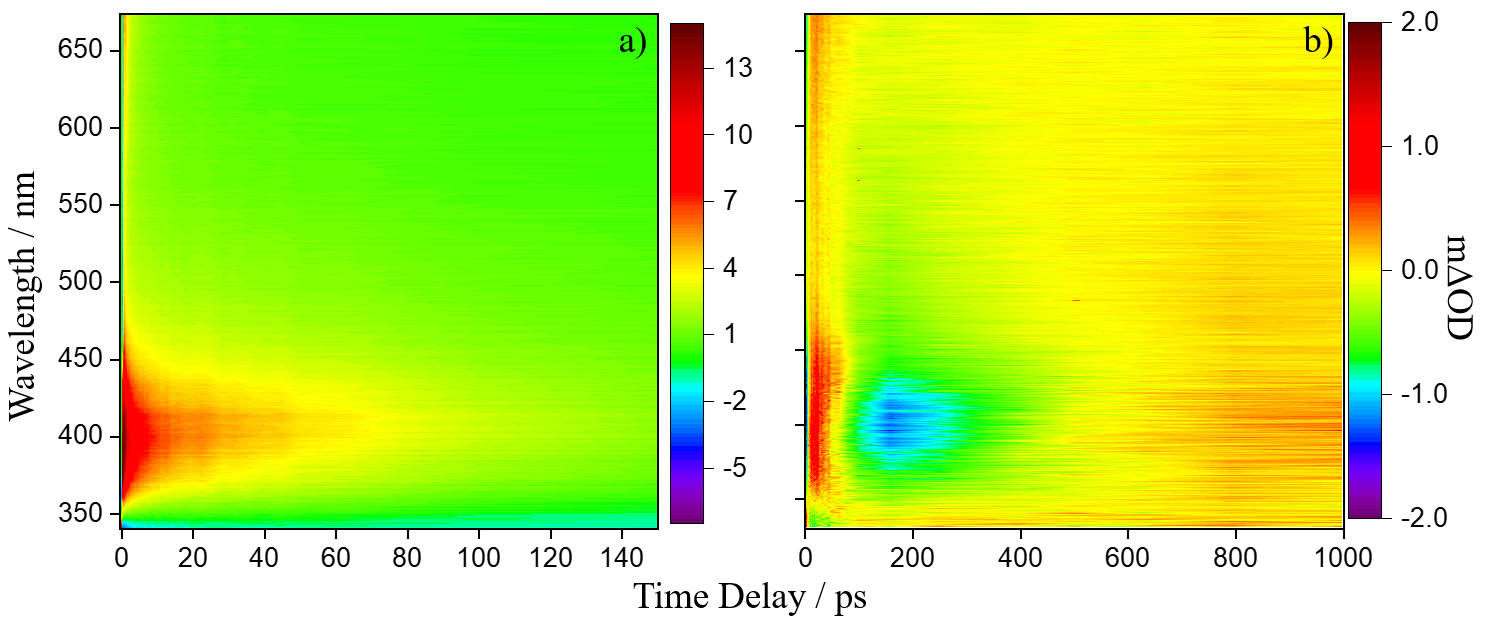


**Figure S7**: a) False colour map of non-normalized transient absorption spectra of **Me-DHDES** in ethanol and b) the corresponding fit residual.

# ^1^H-NMR Spectra of DHDES Pre- and Post-Irradiation


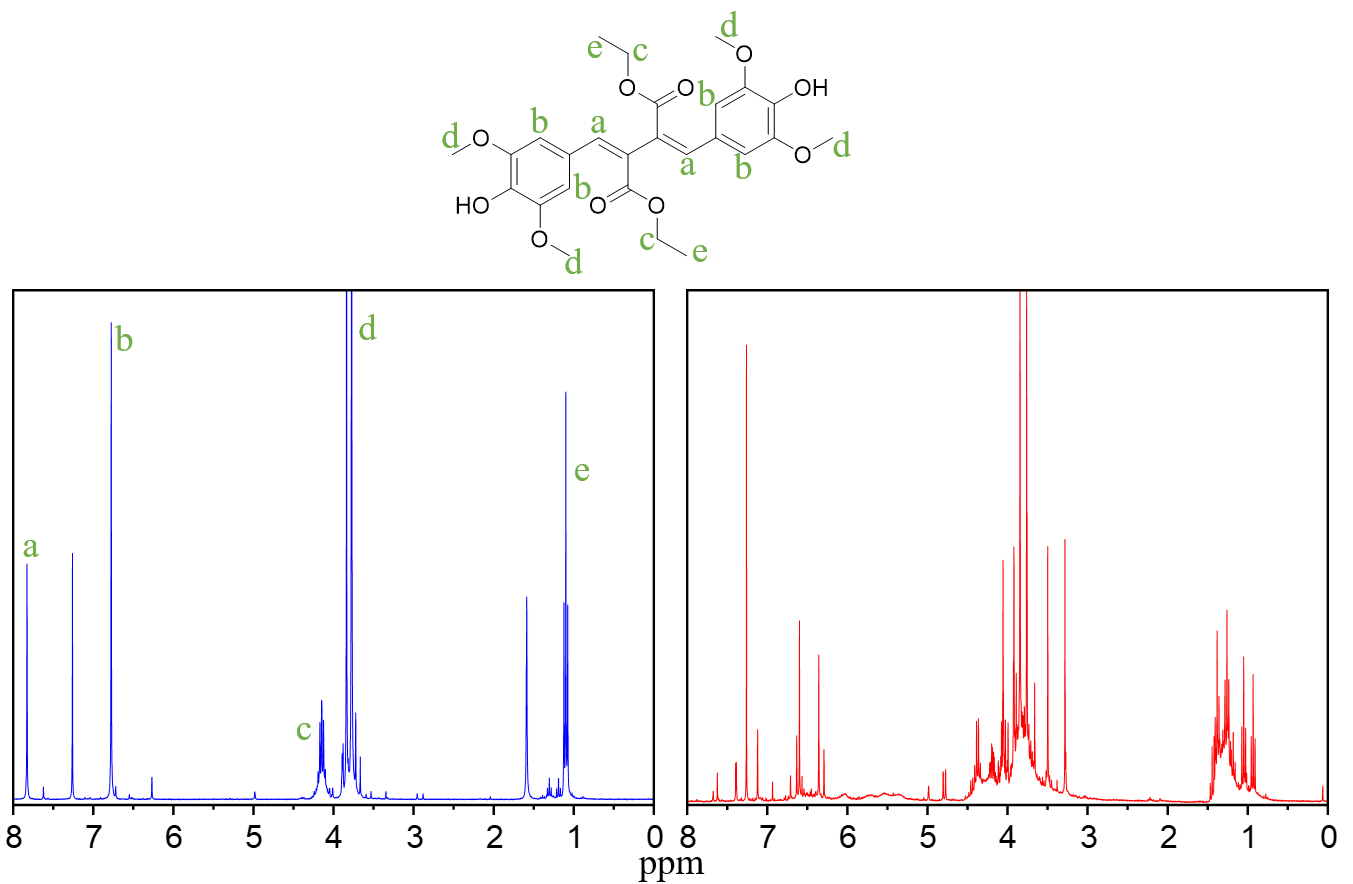


**Figure S9**: ^1^H NMR spectra (CDCl_3_, 300 MHz) of **DHDES** pre (blue) and post (red) irradiation in ethanol. Note that peak b is denoted as A in the manuscript Fig 3.

# Steady-State Irradiation UV/Vis and ^1^H NMR spectra of Me-DHDES


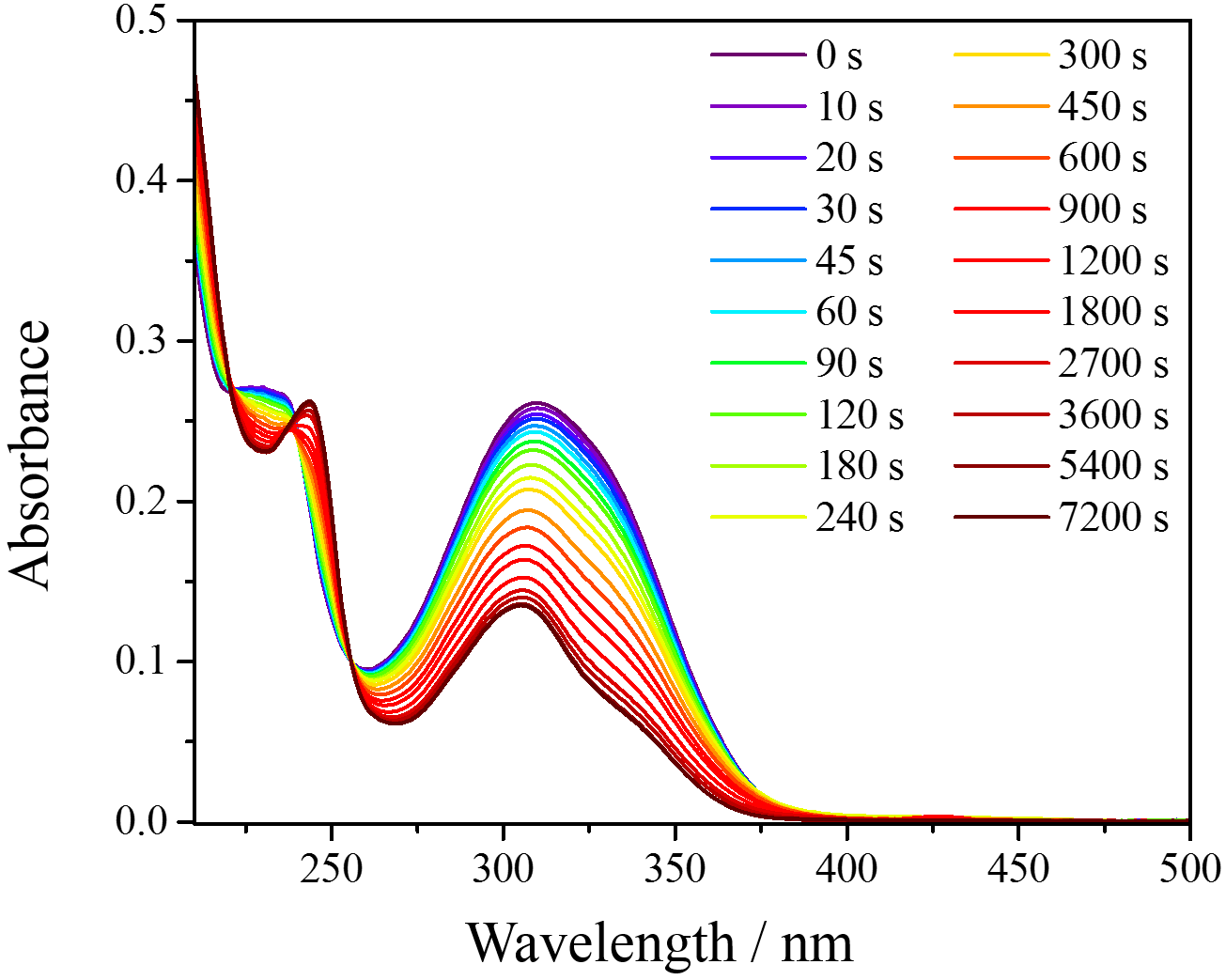


**Figure S10**: UV/Vis spectra of **Me-DHDES** in ethanol (~1 μM) taken at various time points during irradiation at 310 nm over 7200 s.


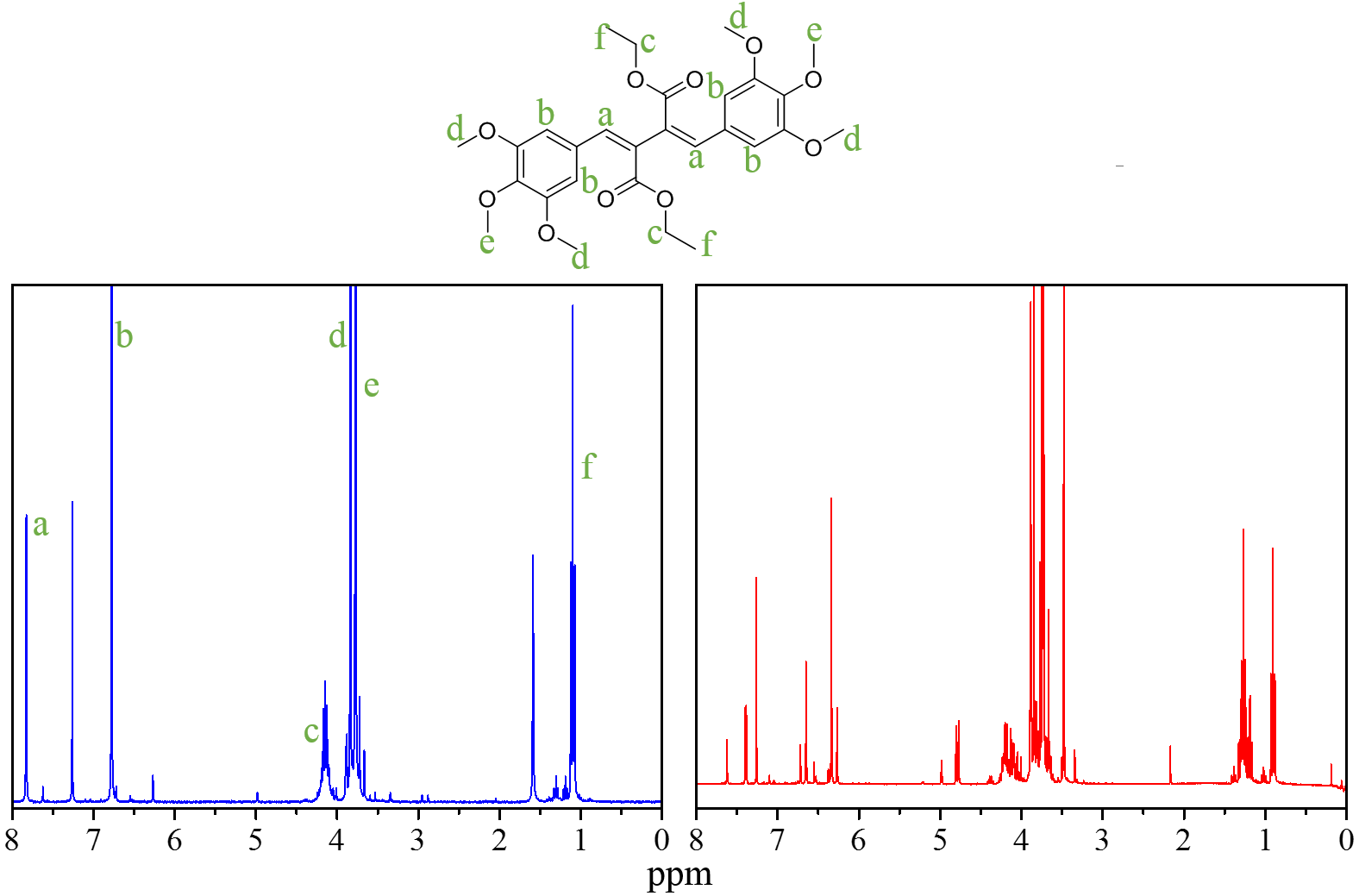


**Figure S11**: ^1^H NMR spectra (CDCl_3_, 300 MHz) of **Me-DHDES** pre (blue) and post (red) irradiation in ethanol.

# Comparison of the EADS of DHDES and ES


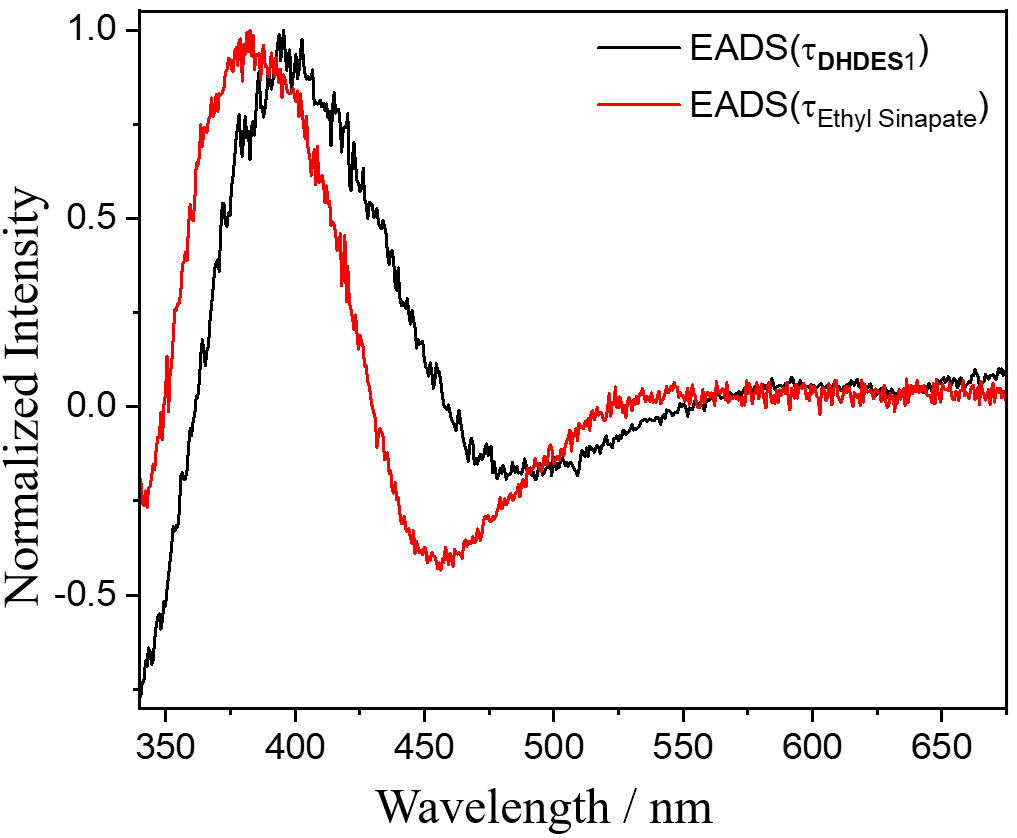


**Figure S12**: The normalized EADS(τ**_DHDES_**_1_) of **DHDES** (black line) in ethanol is overlaid with the normalized EADS(τ_ethyl sinapate_) of ethyl sinapate (red line) in ethanol.


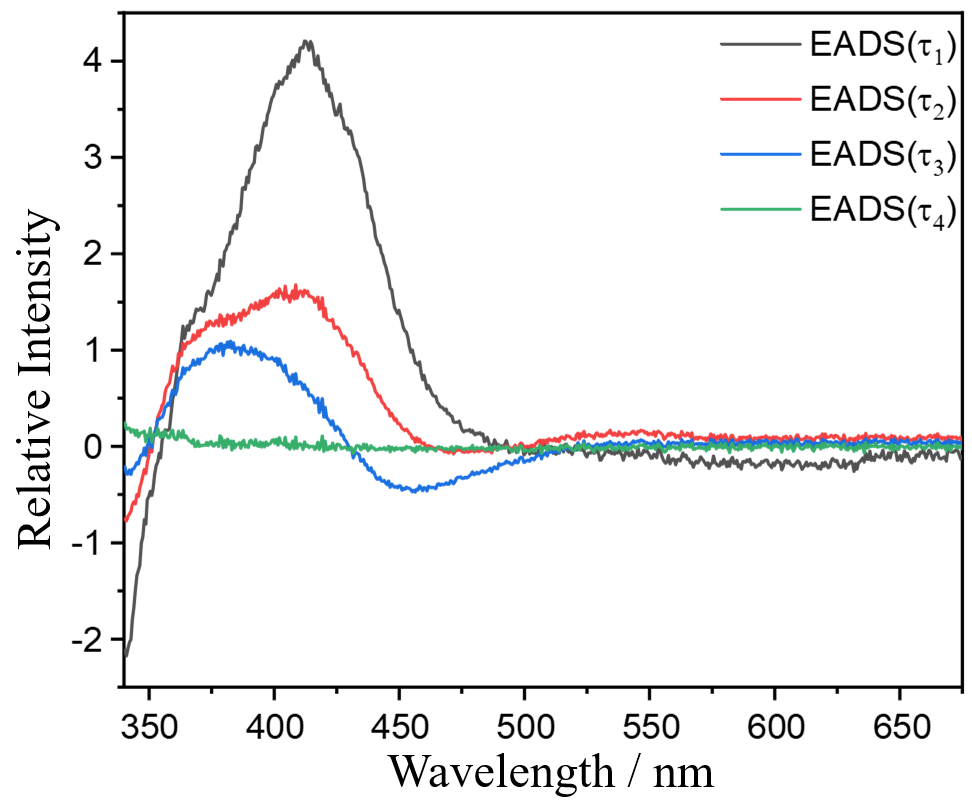


**Figure S13**: The EADS of ethyl sinapate in ethanol, figure adapted from Ref 1

# 1,4-disyringol-1,3-butadiene


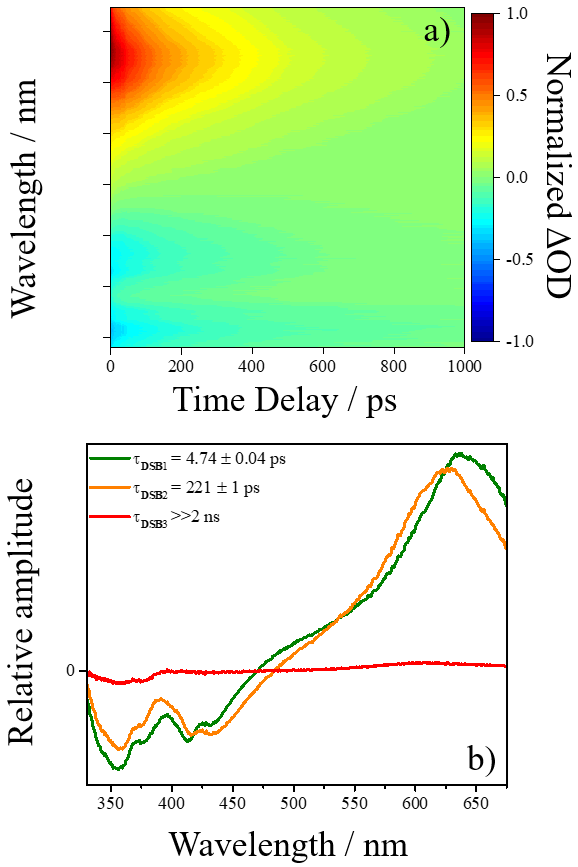


**Figure S14**: a) False colourmap of the transient absorption spectra of DSB with intensity representing normalized change in optical density (OD). b) Additionally, the EADS produced by the sequential global fitting of the transient absorption spectra are also displayed.

# References

**Figure S16**: Calculated ground state structures of *trans,trans*-, *trans,cis*-, and *cis,cis*-isomers of DSB. The relative energies of *trans,cis* and *cis,cis* to *trans,trans* are 0.19 eV and 0.42 eV, respectively.


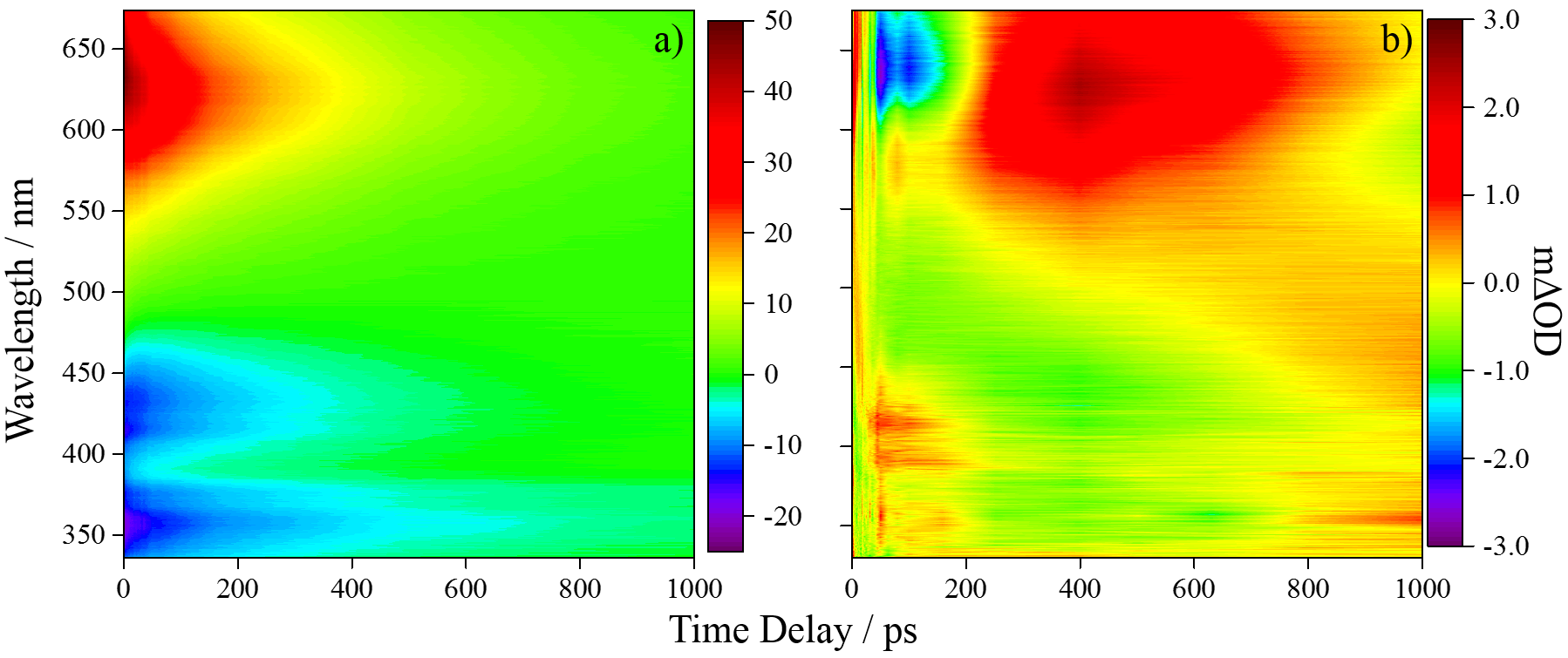


**Figure S15**: a) False colour map of non-normalized transient absorption spectra of DSB in ethanol and b) the corresponding fit residual.

Horbury, M., Flourat, A.L., Greenough, S.E., Allais, F., and Stavros, V. (2018). Investigating isomer specific photoprotection in a model plant sunscreen. *Chemical Communications* 54**,** 936-939.
